# Combination of genomic approaches with functional genetic experiments reveals two modes of repression of yeast middle-phase meiosis genes

**DOI:** 10.1186/1471-2164-11-478

**Published:** 2010-08-17

**Authors:** Michael Klutstein, Zahava Siegfried, Ariel Gispan, Shlomit Farkash-Amar, Guy Zinman, Ziv Bar-Joseph, Giora Simchen, Itamar Simon

**Affiliations:** 1 Department of Microbiology and Molecular Genetics, The Institute for Medical Research - Israel-Canada, The Hebrew University-Hadassah Medical School, Jerusalem, 91120 Israel; 2 Department of Genetics, The Hebrew University, Jerusalem, 91904 Israel; 3 Telomere Biology Laboratory, Cancer Research UK, 44 Lincoln's Inn Fields, London WC2A 3PX, UK; 4 School of Computer Science, Carnegie Mellon University, Pittsburgh, PA 15213, USA

## Abstract

**Background:**

Regulation of meiosis and sporulation in *Saccharomyces cerevisiae *is a model for a highly regulated developmental process. Meiosis middle phase transcriptional regulation is governed by two transcription factors: the activator Ndt80 and the repressor Sum1. It has been suggested that the competition between Ndt80 and Sum1 determines the temporal expression of their targets during middle meiosis.

**Results:**

Using a combination of ChIP-on-chip and expression profiling, we characterized a middle phase transcriptional network and studied the relationship between Ndt80 and Sum1 during middle and late meiosis. While finding a group of genes regulated by both factors in a feed forward loop regulatory motif, our data also revealed a large group of genes regulated solely by Ndt80. Measuring the expression of all Ndt80 target genes in various genetic backgrounds (WT, *sum1Δ *and MK-ER-Ndt80 strains), allowed us to dissect the exact transcriptional network regulating each gene, which was frequently different than the one inferred from the binding data alone.

**Conclusion:**

These results highlight the need to perform detailed genetic experiments to determine the relative contribution of interactions in transcriptional regulatory networks.

## Background

Many biological processes are regulated at the level of transcription. Effector genes with specific roles in biological processes are activated and shut down by transcriptional activators and repressors. The strategy of using such transcription factors to amplify a signal to many target genes is conserved in evolution, and many examples for such regulation are known from yeast to human.

In many cases, specific interactions between transcription factors influence the fate of the process as a whole (some examples are the action of several transcription factors in the Notch signaling pathway, [1], and the activity of the YY1 transcription factor, [2]). Thus, in order to understand a biological process, it is vital to understand the interplay between the transcription factors that govern the process. Very often, transcription networks are very complex, and it becomes difficult to understand the interaction between the transcription factors. To simplify the picture, model organisms with simpler transcription networks are used, of which budding yeast *Saccharomyces cerevisiae *is most useful.

In budding yeast, meiosis has been extensively used as a model for complex developmental processes. The transcriptional control of meiosis in budding yeast is of special interest, since it is composed of several transcriptional waves, controlled by different transcription factors, and has therefore been studied extensively [3]

In yeast, meiosis initiates in diploid cells upon exposure to medium lacking a fermentable carbon source and nitrogen. Typically, meiosis is completed in the formation of a rigid ascus that contains four spores (meiotic products), surrounded by a spore wall.

Budding yeast meiosis was shown to consist of three distinct phases, each characterized by a different set of transcripts [4], [5]. Upon meiosis induction, early phase genes are activated within minutes. The promoter regions of many of these early genes contain a common binding site (termed URS1), which is closely associated with the transcription factor Ime1 [6], [7], [8]. Originally, Ime1 was thought to work together with Ume6 in early meiosis; however, recent work has shown that Ume6 is sent to degradation at this stage and thus cannot participate in early meiotic regulation [9]. Early phase of meiosis extends to the pachytene checkpoint, when homologous chromosomes are aligned after having recombined with each other.

Middle and late genes are transcribed during the meiotic divisions and transcription continues through the formation of the rigid ascus wall [4]. Analysis of promoters of middle meiotic genes revealed that they share several elements, which might be binding sites for transcription factors [10]. Later, the transcriptional activator of many middle meiotic genes, Ndt80, was discovered, and its binding site was identified [11].

Several genome-wide expression studies have been performed on meiotic yeast cultures [12], [13], [14]. These studies have both confirmed and extended classical studies of yeast meiosis. Many more genes were grouped into the previously defined temporal categories, confirming the identity and mode of action of meiosis transcriptional regulators.

Middle meiosis is tightly regulated. Once a cell has passed the pachytene checkpoint and entered middle meiosis it is committed to the meiotic process [15], [16]. Moreover, at this stage the temporal variability between cells is reduced to a minimum and all cells that have started meiosis proceed in a synchronized manner [17]. It has been suggested that this tight regulation and the transient expression of the middle phase transcripts is achieved through the interplay between the transcriptional activator Ndt80 and the repressor Sum1 [18]. Ndt80 was shown to be essential for entry into meiotic divisions, and in its absence cells arrest at the pachytene stage [19]. Ndt80 is induced in early meiosis by the Ime1 transcriptional activator [20] and its activation is facilitated by phosphorylation by Ime2 (a meiosis specific kinase) [21], [22]. Ndt80 binds and activates promoters of genes containing the MSE (middle sporulation element) sequence [12]. Sum1 is a repressor that is associated with the Hst1 histone deacetylase and represses the transcription of many middle phase genes during vegetative growth [23] and during early meiosis phase [20]. Sum1 protein levels fluctuate during meiosis, decreasing prior to entry into meiosis I and increasing after meiosis II [24]. The expression of several Sum1 target genes (such as *SMK1 *and probably also *NDT80*) is deregulated in *sum1Δ *cells, and their expression levels remain high both in early and late meiosis phases [24], [20]. However, no meiotic phenotype has been observed in this *sum1Δ *deleted strain. Sum1 binds a DNA binding site which resembles, and partly overlaps, the binding site of Ndt80 [23], [18]. In vitro experiments have suggested that both transcription factors compete for binding on target promoters [18]. Taken together, it has been suggested that the tight transcriptional regulation during middle meiosis is achieved through competition between Ndt80 and Sum1.

Although this model is possible, it has not been shown to operate in vivo. Additionally, it is not known which genes are regulated by both factors, and to what extent Sum1 is active and necessary in late meiosis.

Here we use chromatin immunoprecipitation coupled with hybridization to genomic DNA microarrays (ChIP-on-chip), together with expression profiling, to determine the complete set of targets of Ndt80 and Sum1 in middle/late meiosis. These data, together with genetic experiments, challenge the generality of the competition model and suggest that Sum1's role in late meiosis may be achieved, in a great part, through its down-regulation of *NDT80*. Our data also help to decipher the transcriptional network during middle/late meiosis. We show that a feed-forward loop governs this network and we analyze the network structure in different transcriptional scenarios. The study may thus be used as a model study for more complex transcriptional networks.

## Results

### Re-establishment of repression of middle phase genes at late meiosis depends on Sum1

**Figure 1:**
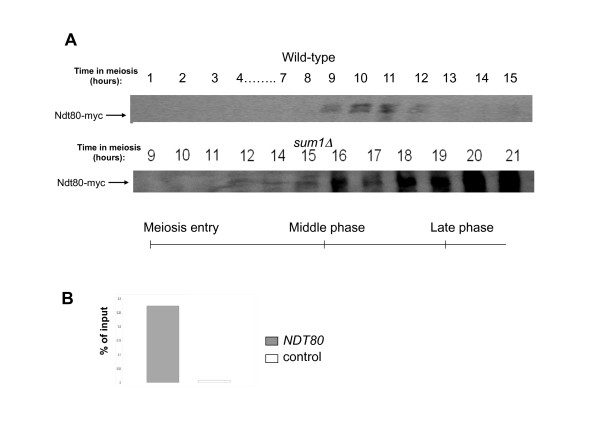
**Sum1 is important for *NDT80 *repression**. **A: **Western Blot of Ndt80-myc during meiosis in WT and *sum1Δ *strains. Note the high levels of Ndt80 in late meiosis in the *sum1Δ *strain. Meiosis Stages are indicated below the blots. **B: **Results of ChIP-PCR on Ndt80-myc for the promoter of *NDT80 *in *sum1Δ *cells at 15 hours in sporulation medium. Ndt80 binds to its own promoter when Sum1 is absent.

In Yeast meiosis, the middle phase genes are induced upon activation of the Ndt80 transcriptional activator. Many of the Ndt80 induced genes are consequently repressed at the late meiosis phase [24]. This late phase repression was attributed to a competition between Ndt80 and the transcriptional repressor Sum1, which is upregulated at late meiosis [24]. According to this view, Sum1 is essential for the repression of the middle genes. However, deletion of *SUM1 *in the SK1 background does not cause a significant increase in the levels of *NDT80 *and other middle meiosis genes at late stages of meiosis [24], suggesting that other factors beside Sum1 may be involved in the repression of middle genes in SK1 background. On the other hand, in W303 background, Sum1 seems to play an important role in late meiosis repression. Deleting *SUM1 *in W303, causes accumulation of Ndt80 protein (a classical middle meiosis gene) in late meiosis phase in a sharp contrast to the transient middle meiosis expression pattern characterizing Ndt80 in WT W303 cells (Figure 1a). This aberrantly expressed Ndt80 maintains its DNA binding activity and in the *sum1Δ *strain its binding to its own promoter (a known target, [11]) can be detected even at 15 hours after switching to sporulation medium (Figure 1b).

### Subtle defects in meiosis in the absence of Sum1

**Figure 2:**
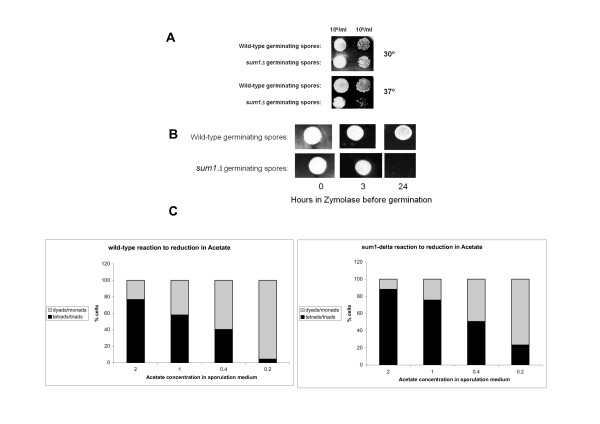
**meiotic phenotypes of *sum1Δ *cells**. **A**: The growth resulting from drops of germinating spores of wild-type and *sum1Δ *strains were compared at two temperatures. *sum1Δ *spores are more heat sensitive than the spores of the wild-type strain. **B: **Drops of germinating spores of wild-type and *sum1Δ *strains which were incubated with Zymolase for indicated times. After treatment with Zymolase, *sum1Δ *spores do not germinate, while wild-type cells still do. **C: **percentage of asci with 3-4 spores and asci with 1-2 spores in different acetate concentrations in the sporulation medium. The results are compared between wild-type and *sum1Δ *meiosis. *sum1Δ *cells do not react as well as wild-type cells to the reduction in acetate concentration, always keeping a lower amount of asci with 1-2 spores.

The important role of Sum1 in late meiosis in the W303 background is further emphasized through an analysis of the phenotypes of the *sum1Δ *strain. In contrast to the SK1 background in which no phenotype was detected in the *sum1Δ *strain [24], in W303 background we observed meiotic defects. Analysis of the sensitivity of spores to heat and to Zymolyase digestion, revealed clear differences between WT and *sum1Δ *spores. We found that the germination of spores resulting from the *sum1Δ *meiosis (strain MKsumdel) is heat sensitive (Figure 2a). We also found that after 24 hours in Zymolyase, an enzyme with lytic activity against yeast cell walls (but not spores), the *sum1Δ *spores did not germinate at all, whereas wild-type spores germinated efficiently (Figure 2b). These findings suggest that the spore wall of spores arising from *sum1Δ *meiosis might be defective.

Yeast cells undergoing meiosis react to restricted energy (low levels of acetate) by producing fewer spores per ascus, 1-2 instead of 4 [25]. When we monitored the energy management in *sum1Δ *meiosis by counting the number of spores per ascus at different concentrations of acetate in the sporulation medium (see Methods), we found that *sum1Δ *sporulation did not respond to acetate restriction as well as the wild-type strain did. *sum1Δ *cells undergoing sporulation produced a lower frequency of asci with 1-2 spores than wild-type cells (Figure 2c). This result hints to a further defect in spore wall synthesis control, or to a more general energetic defect in these cells, that fail to react to environmental cues in the prescribed optimal manner.

We conclude that in W303 strains, Sum1 is an important repressor, which is responsible for the re-establishment of repression of middle phase genes towards the late phase of meiosis. In the absence of Sum1, defects occur in spore formation, which lead to defects in spore germination.

### Analysis of expression profiling

**Figure 3:**
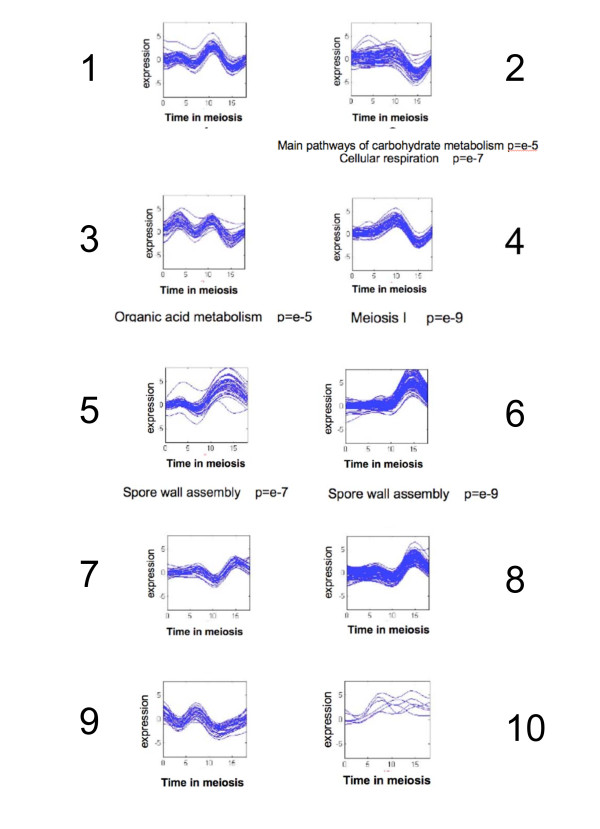
**Clustering of RNA expression analysis in W303 meiosis**. K mean clustering results on the de-convoluted W303 expression data (see Methods). The expression values (log_2 _ratios) along the meiosis are shown for all the genes divided into ten clusters. GO categories enrichments are shown beneath the graphs.

To gain better insight into middle and late phase meiotic gene expression in yeast of W303 genetic background and its relation to Ndt80 and Sum1 activity, we performed an expression profile experiment extended to include the late meiosis phase (previous experiments with the W303 strain last only 12 hours in sporulation medium [13] while our profile extends to 18 hours in sporulation medium). The expression profile experiment was designed to pay special attention to the asynchronous nature of meiotic cultures (see Methods). To do so, we used an algorithm for the de-convolution of gene expression data [26], [27] by using DAPI staining information for estimation of the stage of meiosis achieved at every time point (see Methods). The raw expression data was therefore corrected to reflect the gene expression of a synchronous population of cells. The results of the expression analysis are detailed in Additional file 1. Clustering (see Methods and Additional File 1, validation of results by RT-PCR is presented in Additional file 2) of the expression data reveals ten clusters of genes with different temporal expression patterns (Figure 3 and Additional File 1). Two of the clusters (clusters 5 and 6) show a distinct induction during middle meiosis, and were enriched for sporulation and spore wall synthesis GO categories. These two clusters together contain 138 genes, of which 78% (108 genes) were identified as middle meiosis genes by at least one of the previous methods (expression upon Ndt80 over-expression [12], expression during middle meiosis (clusters 4-7 in [13]), and the existence of Ndt80 binding sites [28]). Among the 30 middle meiosis genes that were not identified before by genomic methods, we found genes encoding important meiotic cell cycle regulators such as Clb1 ([29]) and Hos4 (part of the Set3 complex [30]), and other cell cycle regulators such as Apc9 and Cdc37. Our analysis reveals for the first time a cluster of 50 genes (cluster 2) that shows distinct repression during middle meiosis. This cluster is highly enriched for genes involved in metabolism, suggesting a possible role for repression of respiration and metabolism towards the advanced stages of yeast meiosis.

### Binding profiles of Ndt80 and Sum1 during meiosis middle and late phase

To understand the control of middle meiotic genes by Ndt80 and Sum1, we performed ChIP-chip experiments with these factors in meiosis. According to our Western-blot analysis (Figure 1a), and previous studies [12], Ndt80 is expressed exclusively at middle meiotic time points. To understand which genes it regulates, ChIP-chip was performed on samples collected 9 hours after transfer to sporulation medium (see Methods). Using a stringent threshold (false discovery rate (FDR) = 0.05; see Methods) we found 302 Ndt80 target genes (Additional file 1). Three lines of evidence suggest that these genes are indeed Ndt80 targets -First, the list of Ndt80 targets is highly enriched for several main meiotic GO categories - spore wall assembly (p = 1.4e-10) and sporulation (p = 1.6e-8), and is also enriched for the cell division GO category (p = 0.0018), a mitotic related category, intimately associated with meiotic divisions as well. Second, using de novo motif search algorithms (WebMotifs; http://fraenkel.mit.edu/webmotifs/ and [31]), we found that the promoters of these genes are highly enriched for the presence of the middle sporulation element (MSE) (specificity score 10^-29^), the known binding site of Ndt80 [11], [18]. Finally, we detected binding of Ndt80 to about half of its previously suggested targets (51 out of 98 targets suggested by Wang *et al *[28], based on bioinformatic considerations).

Moreover, analysis of published meiotic expression profiles revealed that our ChIP-on-chip data captured roughly a fifth (71/330) of the genes that belong to previously identified middle phase clusters (cluster 4-7, Primig *et al*, [13]), and almost half (65/142) of the genes in one of these clusters (cluster 5, Primig et al.), strongly supporting the role of Ndt80 binding in middle meiotic gene expression. In our meiotic expression profile, Ndt80 was found to bind the promoters of 45/138 (32%) genes belonging to the middle meiotic clusters 5 and 6.

To find out which promoters Sum1 binds in the late phase, we performed a ChIP-chip experiment on Sum1 at 15 hours after transfer to sporulation medium. Using a threshold similar to the one used for Ndt80 (see Methods), we detected 479 Sum1 targets (Additional file 1). The list of targets is similar but not identical to ChIP-chip on Sum1 in mitotic cells previously performed [32]. Most, but not all mitotic targets were found to be bound in meiosis (73.5%, 89/121), and many specific late meiotic targets have been found (390 promoter regions). This list of genes is significantly enriched for the main meiotic GO categories - spore wall assembly (p = 2.6e-9), and sporulation (p = 3.9e-8) and for the cell division category (p = 0.00045), the same categories enriched for in the Ndt80 binding data. Motif analysis found enrichment for the MSE* motif (specificity score 10^-18^) which is the known binding site of Sum1 [18], [3]. Here also our experimental data found direct evidence for the interaction of Sum1 with the promoter regions of most of its putative target genes (61 out of 77 targets suggested by Wang *et al *[28]).

**Figure 4:**
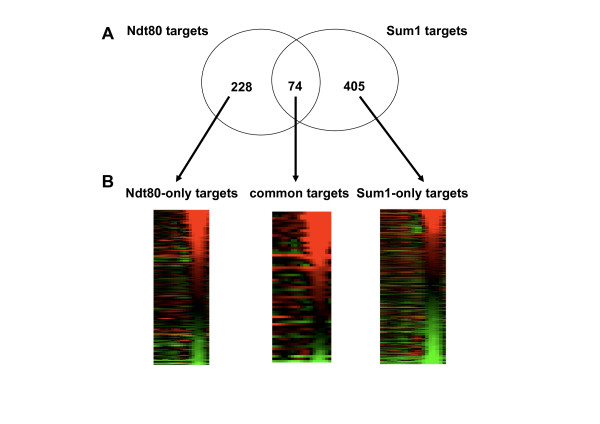
**Ndt80 and sum1 targets classification**. **A**: A Venn diagram capturing the overlap between Ndt80 and Sum1 targets. The numbers indicate the number of targets in each category. **B**: For each category of genes a heat map capturing the expression profile during meiosis is shown. a significant fraction of the Ndt80-only and the common categories genes have a middle meiosis expression pattern (15% and 25% respectively), whereas only few such genes (3%) were in the Sum1-only group. Note the resemblance between the expression patterns of the Ndt80-only targets and the common targets.

Further analysis revealed three groups of target genes (see Methods for binding thresholds): i) Ndt80-only targets, ii) common targets of both regulators, and iii) Sum1-only targets (Figure 4 and Additional file 3). These assignments were confirmed by performing ChIP-PCR on several representative genes (Additional file 4). Binding site analysis further confirmed the categorization. Scanning the target promoters, using a threshold that recognizes binding sites in 5% of the promoters (see Methods) revealed a 2.6 fold enrichment for the MSE motif (the Ndt80 binding motif) in the Ndt80-only targets and a 2.2 fold enrichment for the MSE* motif (the binding motif of Sum1 [18]) in the Sum1-only targets. On the other hand only the common targets were enriched for both motifs (4.6 and 4.8 folds enrichment for MSE and MSE*, respectively). Further support for the distinction between these groups came from the Sum1 binding data in mitotic cells [32]. While 25% (19/74) of the common targets were also identified by Harbison *et al *[32] as Sum1 targets (p < 0.001), only 0.9% (2/228) of the Ndt80-only targets group were identified as Sum1 targets. Furthermore, Pierce *et al *[18] have identified the genes that are derepressed (> 2 fold) in rich medium upon *SUM1 *deletion. In the common targets group 17.5% (13/74) were derepressed compared to only 4.6% (10/218) in the Ndt80-only group. It should be noted that a similar percentage of the genes in both groups was affected (> 2 fold) upon over-expression of Ndt80 in rich medium (42%, 31/74 in the common targets and 34% (78/228) of the Ndt80-only targets [12]).

To further characterize the three groups we performed GO annotation analysis. This analysis revealed that only the common targets group is significantly enriched for the major meiotic categories - sporulation (p = 6.2×10^-13^) and Spore wall assembly (p = 1.02×10^-12^), and for the cell division category (p = 2.92×10^-7^), whereas the other groups are populated by genes involved in multiple functions related to meiosis such as RNA metabolism and transport but are not significantly enriched for any specific GO category (data not shown). It thus seems that Ndt80 and Sum1 bind to a common set of targets, which is functionally important for middle meiosis, and has a middle meiotic expression pattern. In addition, these two factors each bind to a unique set of targets as well.

In spite of the apparent differences in the regulation of the common targets and the Ndt80-only groups, they show a surprisingly similar middle-meiotic expression pattern, being induced in middle phase, and re-establishing repression at the late phase (Figure 4).

**Figure 5:**
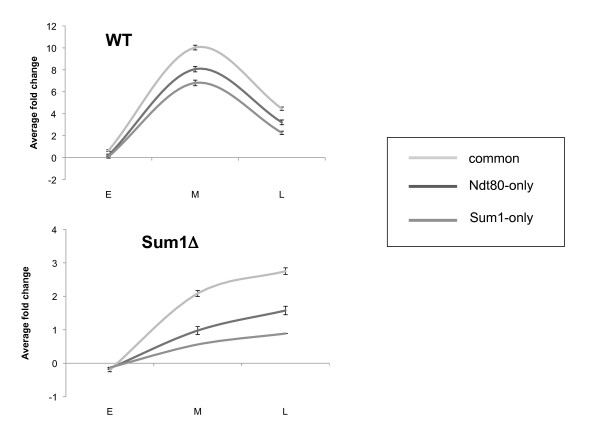
**expression pattern of middle meiosis genes in *sum1Δ *meiosis**. Expression (fold change over WT strain grown in rich medium) of all middle meiosis genes (clusters 5 and 6) separated into three categories (common targets - light gray, Ndt80 only targets - black, Sum1 only targets - dark gray) in WT and in *sum1Δ *strains. Average and standard error of the expression at three time points (8, 14 and 18 hours in WT and 10, 17 and 21 hours in the *sum1Δ *strain) are shown.

In order to decipher the source of late meiosis repression of the Ndt80 only target genes, we performed a genomic expression profile on *sum1Δ *cells in early, middle and late meiosis (raw data presented in Additional File 1, validation of results by RT-PCR is presented in Additional File 2). This profile clearly shows (Figure 5), that the RNA of both the common and the Ndt80-only targets accumulates without repression in *sum1Δ *cells, suggesting an involvement of Sum1 also in the down regulation of Ndt80-only targets. This explanation of the results is preferable over the assumption that most of the cells in the *sum1Δ *strain are stuck in a middle meiosis stage, since this strain can complete sporulation and form normal looking asci. Moreover, analysis of the expression profile data revealed that most late meiotic genes, which are not targets of Ndt80 or Sum1, are expressed also in the sum1Δ strain (there are 78 genes that are expressed in late meiosis (cluster 8 Figure 3) and are not bound by Ndt80 or Sum1. 64% of them are expressed in late meiosis also in the sum1Δ strain). It is noteworthy that the level of expression of all targets is relatively low in the *sum1Δ *strain compared to wild type. This might hint to a positive role mediated indirectly by Sum1 on the expression of meiotic genes, possibly through interaction with the meiotic early phase transcription regulators.

### Control of the *NDT80 *promoter by Sum1

**Figure 6:**
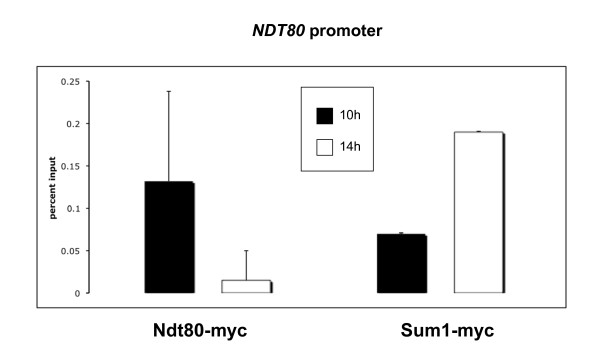
**Ndt80 and Sum1 bind at different times to the *NDT80 *promoter**. Results of ChIP-PCR for binding of Ndt80-myc and Sum1-myc on the promoter of *NDT80 *at two different time points in meiosis, 10 and 14 hours in sporulation medium. Ndt80 and Sum1 bind the *NDT80 *promoter at different times, Ndt80 at 10 hours and Sum1 at 14 hours. Error bars represent different PCR reactions done on two different ChIPs.

How is the repression of Ndt80-only targets in the late phase established without binding of Sum1? One of the possibilities is that the binding of Sum1 to the *NDT80 *promoter [32] is responsible for the repression of Ndt80 in late meiosis, allowing for the repression of the Ndt80-only targets in an indirect manner. We wanted to check if Sum1 binds the Ndt80 promoter in meiosis late phase. To this end we performed chromatin immunoprecipitation and determined Ndt80 and Sum1 binding to the *NDT80 *promoter in two time points by PCR. We found a shift in the transcription factor binding along meiosis. While Ndt80 occupation decreases between 10 and 14 hours in sporulation medium, Sum1 increases its occupancy on the *NDT80 *promoter (Figure 6). These results suggest that the regulation of the *NDT80 *gene is accomplished through the binding of Ndt80 to its promoter during middle phase, which is later replaced by Sum1, thus ensuring the down-regulation of *NDT80 *in late meiosis.

### Ndt80-only targets are repressed indirectly while common targets are also affected by Sum1 direct binding

Our finding of Sum1-dependent down regulation of Ndt80-only targets suggests that Sum1 affects the transcription of those genes indirectly through its regulation of the *NDT80 *promoter [11]. On the other hand, regulation of the common target genes can occur both directly, through the binding of Sum1, and indirectly, through its regulation of *NDT80*. To check which of the modes of control is the predominant one, we interfered with the binding of Sum1 to the *NDT80 *promoter. To this end we used a yeast strain in which the *NDT80 *promoter was replaced by an inducible promoter (MK-ER-Ndt80) that can be turned on by administration of Estradiol to the medium (see Methods and [29]). In this strain, *NDT80 *transcripts accumulate in late meiosis since Sum1 no longer represses it (Additional file 5). Comparison of the repression of different target genes in late meiosis stages in wild-type (WT) and in the MK-ER-Ndt80 strains should allow us to separate between the effects of Sum1 on gene expression through its regulation of *NDT80 *and other effects of Sum1. This strain enters meiosis after the addition of Estradiol in a similar efficiency as a WT W303 strain (Additional file 6) but rarely (< 5%) completes sporulation. Nevertheless, expression profiling of this strain at three time points following the induction of *NDT80 *by estradiol (Additional File 1, validation of results by RT-PCR is presented in Additional File 2), revealed that these cells do enter the late meiotic stages since most (54/78) of the Ndt80 and Sum1 independent late meiosis genes (genes of cluster 8 in Figure 3 which are not bound by Ndt80 or Sum1), are expressed also in this strain.

**Figure 7:**
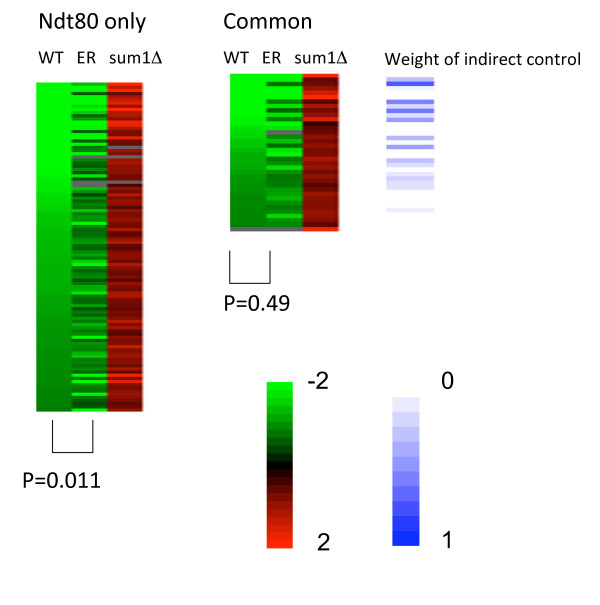
**late phase repression of target genes in a strain with an inducible *NDT80***. repression level of common and Ndt80-only targets (difference between middle and late time points) is presented as a color coded heat-map in WT, Ndt80-ER and sum1Δ backgrounds. Note that in Ndt80-only targets WT and ER samples are significantly different, while in the common targets the repression pattern is similar. Both groups show same level of induction in the sum1Δ strain indicating that the repression of all those genes is Sum1 dependent. The metric (see Methods) capturing the relative contribution of the indirect edge in the repression of the target genes in the common group is shown with a blue color code. Note presence of genes with large weight as well as genes with small weight values in the common targets.

To assess the effect of Sum1 on the repression of Ndt80 target genes, we analyzed the changes in the expression pattern of 140 Ndt80 targets that show late phase repression (in the WT strain, see Methods). For each of those genes we calculated the fold change between the middle and the late time points in the WT, MK-ER-Ndt80 and sum1Δ strains (Figure 7). As expected, the repression of Ndt80-only target genes was compromised in the MK-ER-Ndt80 strain (an average of 1.55 fold versus 1.85 fold in the WT strain; P = 0.011), suggesting that Ndt80 down regulation by Sum1 plays a significant role in the regulation of those genes. On the other hand, the average repression of the common genes was the same in both strains (1.91 fold), suggesting that in those genes the repression is probably not gained through the down regulation of Ndt80 but through the direct interaction of Sum1 with their promoter. Both groups (common and Ndt80-only targets) were similarly affected in the *sum1Δ *strain (Figure 7).

**Figure 8:**
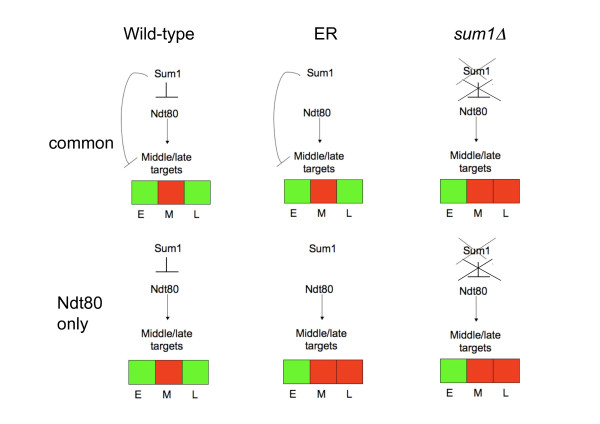
**Model for the regulation of meiotic expression by Ndt80 and Sum1 in different genetic backgrounds**. for every genetic background (WT, ER and sum1-deletion), the structure of the feed forward-loop is presented, as is the expected induction and repression pattern of different targets (common and Ndt80-only targets, in rows, red for induction, green for repression) at three time points along the meiosis: E-early, M-middle and L-late.

Taken together, our results decipher two regulatory mechanisms for middle meiotic gene repression at late meiosis. The repression is achieved either directly, through Sum1 binding to the promoters of the target genes, or indirectly, through Sum1 regulation of the expression of the *NDT80 *gene. Both regulatory mechanisms can act together in the repression of the common genes, forming a feed forward loop regulatory motif (Figure 8). Our detailed genetic analysis allows us to assess the relative contribution of the direct and indirect effects of Sum1 for each of the common genes. By comparing the repression levels in both strains we have calculated a metric (see Methods), which captures the relative contribution of Ndt80 regulation to the repression pattern of each gene (Figure 7 and Additional File 7). We found that approximately 50% of the genes were repressed to the same extent in both strains, suggesting that most of the repression of those genes is achieved independently of Ndt80. The remaining genes were repressed to different levels in the two strains suggesting a different weight for the direct and indirect repressing mechanisms for each gene.

## Discussion

Many biological processes are regulated at the transcriptional level. This mode of regulation gives an evolutionary advantage, as a change in a small number of transcription activators and repressors, investing relatively little energy, can be amplified to affect large-scale biological events. Transcription regulation is frequently achieved through the interplay between activators and repressors.

Meiosis is an example to such a cellular differentiation process. Common to most eukaryotes, meiosis forms haploid gametes from diploid non-differentiated cells. In budding yeast, meiosis has been studied in detail, and several transcriptional regulators of the process have been identified [3]. Whole-genome expression during meiosis was performed by several groups ([12], [13], [14] and this study), confirming previous classical studies of gene transcription during meiosis [4], and revealing an interesting repertoire of regulation patterns of hundreds of genes that are either induced or repressed during meiosis. Of the genes induced during the middle phase of meiosis many show a distinct middle meiosis expression pattern in which transcription is induced and then repressed few hours later (cluster 5, 6 in Figure 3). The transient regulation of these genes has been suggested to occur through competition between an activator (Ndt80) and a repressor (Sum1). Indeed, bioinformatic analysis revealed that the promoters of many of the middle meiosis genes contain binding sites of both transcription factors [18]. The implication of this regulatory model is that the promoters of the middle genes are occupied first by the activator in middle meiosis and then are replaced by the repressor, which shuts down transcription in the late phase. We were able to confirm in vivo for the first time, that this is indeed what happens on the promoter of *NDT80 *itself, on which Ndt80 is bound at middle meiosis (10 hours) and is replaced by Sum1 at later stages (14 hours) (Figure 6). Furthermore, we have found by ChIP-on-chip (Figure 4) a large set of genes commonly bound by Ndt80 and by Sum1. Although we cannot rule out the possibility that both factors are bound simultaneously to these promoters, analysis of Ndt80 and Sum1 protein levels (Figure 1 and Additional File 8) suggests that at 9 hours mainly Ndt80 binds whereas at 15 hours mainly Sum1 is bound to the promoters.

The competition model suggests that Sum1 binding to the promoters is needed for the down regulation of the target genes. Surprisingly, we have observed a similar repression at late meiosis of genes bound only by Ndt80 (Figure 4). Analysis of gene expression of a yeast strain deleted for *SUM1 *revealed that Sum1 is crucial for the down regulation of all kinds of target genes, including Ndt80 target genes that are not bound by Sum1 (Figure 5). This down regulation is probably linked to the fact that Ndt80 itself is down regulated by Sum1 and thus at late meiotic stages its levels decrease in a Sum1 dependent manner (Figure 1, 4 and 6). Indeed, releasing Ndt80 from the control of Sum1 by using a yeast strain containing an inducible Ndt80 (MK-ER-Ndt80 strain) results in losing most of the late meiosis repression in some of the Ndt80 targets (Figure 7). We were able to show that while in the Ndt80-only targets, most of the repression pattern, although Sum1 dependent, is a result of an indirect effect of Sum1, in the common targets both pathways- some genes are repressed mainly directly by sum1, whereas in other genes the indirect repression by Sum1 is also important. Using the MK-ER-Ndt80 strain, we were able to quantify the effects of Sum1's binding to *NDT80 *on the repression pattern in late meiosis phase. We conclude that the interplay between an activator and a repressor in a given process may be complex and may depend on the specific context of the targets bound by both factors. Individual targets may vary therefore in the degree affected by each factor. Genetic manipulation may help to quantify this effect.

Promoters of genes from the common targets group are bound by both Ndt80 and its repressor Sum1. This type of regulatory pattern is abundant in yeast (and in *E. coli*), and was termed a "feed-forward loop" [33], [34] or more specifically a "coherent feed-forward loop" type 2 [35]. This type of interaction was suggested to be suitable for cases in which a biological process is driven in a single direction, a definition that suits middle meiosis stages in which the yeast cells are already committed to meiosis. Studying feed forward loops as a group of network motifs (Reviewed in [36]) assumes that the contribution of the direct and indirect effects of the upstream factor are equal and similar for all genes. However, our results with the MK-ER-Ndt80 strain reveal that this assumption may not always be true. We find that different genes are repressed to different extents in the mutant strain, suggesting that the relative contribution of the direct and the indirect effects of the upstream factor may be different for different target genes. Moreover, our finding that in some genes most of the repression is carried out through the down-regulation of Ndt80, and that the contribution of the direct binding of Sum1 to the target genes is relatively small, demonstrates the importance of measuring the actual contribution of different edges in order to precisely analyze network motifs. In order to quantify the differences between the two regulatory modes of Sum1 (direct and indirect control), we calculated a metric capturing the relative contribution of the regulation of the target genes through *NDT80*. We suggest that introducing such metrics into other transcriptional regulatory networks might prove to be very important for understanding the true nature of the genetic interaction.

The Sum1 dependent down regulation of genes in late meiosis seems to be important for spore endurance, since the spores of a *sum1Δ *deletion strain are more susceptible to heat shock, Zymolase treatment and sporulation in a limiting carbon source medium (Figure 2). In contrast to our results in W303 genetic background, previous analysis of *sum1Δ *sporulation in SK1 background did not reveal any severe meiotic phenotype [24]. In spite of the lack of an obvious meiotic phenotype upon *SUM1 *deletion, a severe meiotic phenotype was observed when the *SUM1 *deletion was combined with deletion of components of the pachytene checkpoint [24]. This observation supports our assumption that additional players are involved in late phase repression in SK1. Different QTLs contributing to the different sporulation efficiency of SK1 and S288C (a strain close to W303) have already been found in a previous study from our lab [37]. Further experiments are needed to determine if there is a connection between these QTLs and the involvement of different repressors in late phase transcriptional repression.

## Conclusions

By a combination of genetics and genomics experiments, we show an active interplay between two transcription factors on a subset of common targets during the middle and late phases of yeast meiosis. This interplay is responsible for the expression patterns of many genes during those stages, and thus is responsible for the biological outcome of these meiotic stages. We show that the absence of one of the factors from the meiotic cells has deleterious phenotypic consequences, and changes the expression patterns of these transcription factors' targets. We show that in middle-late meiosis the expression pattern is regulated primarily through the regulation of the *NDT80 *transcript levels. This reminds one of the regulation of the early phase in meiosis of *S. cerevisiae*, much of which is regulated by the *IME1 *transcript levels [38]. Having two master regulators for the two major parts of meiosis is of great regulatory importance, as the first master regulator is in charge of the uncommitted phase of meiosis, and the second for the committed phase of meiosis [16]. A separation between two regulatory phases in meiosis, one before the meiotic divisions and the second one starting at the meiotic divisions might be conserved in evolution. For example mei4, a transcriptional regulator from *Schizosaccharomyces pombe*, regulates the second phase in meiosis that starts at the meiotic divisions [39], [40], similar to Ndt80.

## Methods

### Strains

All strains used in this study were of W303 background (a list of the strains appears in Additional File 9).

We used the Ndt80-myc (Z1615) and Sum1-myc (Z1613) strains from Harbison *et al *[32] and transformed them with a plasmid containing the genes *HO *and *URA3 *[41]. The transformants were allowed to switch mating type, mate and homozygous diploids were picked and tested for sporulation and mating [37]. Subsequently, the plasmid was deliberately lost by growth of diploids on 5FOA containing medium. Tagging of Ndt80 and Sum1 did not affect sporulation efficiency or timing, as compared to W303 wild-type cells (data not shown).

A diploid Ndt80-myc *sum1Δ *deletion strain was made by two steps: first, the *SUM1 *ORF was replaced with *KANMx *in a haploid W303 Ndt80-myc strain, using homologous-recombination based transformation [42]. Next, the *HO-URA3 *plasmid was transformed into this *sum1Δ *strain and transformants were allowed to switch mating type and form diploids, as described above.

The strain MK-ER-Ndt80 (inducible Ndt80 by estradiol, see Carlile *et al *[29]), was made by inserting a fragment of the plasmid PKB80 into the *ura3 *locus (selecting for Ura+ transformants). The plasmid contains the genetic construct P_GPD1_-*GAL4*(848).ER::*URA3 *(constitutively transcribed *GAL4.ER *by the *GPD1 *promoter). After integration to the genome [42], the nuclear retention of the Gal4 transcriptional activator was dependent on the presence of estradiol. To create the estradiol regulated *NDT80*, the construct TRP1::pGAL-*NDT8*0 was inserted by transformation [41] into the ORF of *NDT80*. The plasmid and construct were received from Angelica Amon's lab, MIT. The cells of this strain were incubated in SPII medium for 8 hours, when L-estradiol (Sigma) was added to the medium at a concentration of 1 μM. Upon such induction of *NDT80*, the cells underwent meiosis (Additional File 6), but did not complete sporulation (< 5% asci were detected).

### Media and sporulation conditions

For standard growth we used rich medium (YPD). For sporulation, we grew diploids in GNA medium supplemented by 2% glucose at 30°for 18 hours to a concentration of 2 × 10^7 ^cells/ml as described [13]. Cells were spun, washed with water, and incubated in SPII sporulation medium at a concentration of 2 × 10^7 ^cells/ml at 30°with vigorous shaking, as described [13]. The low acetate concentration experiment was done by diluting the initial acetate concentration (20 gr/L) to the concentrations indicated in Figure 1 with DDW, as described [43].

### Western blot analysis

Equal amounts of protein extracted from cells at various time points along meiosis were applied to a polyacrylamide gel and Western blot analysis was performed as described [44], using mouse anti myc 9E11 monoclonal antibody (Santa-Cruz Biotechnology, http://www.scbt.com) as a primary antibody. We validated the uniform loading using Ponceau S staining (data not shown) since the levels of housekeeping genes (such as actin genes) are not uniform during meiosis.

### DAPI staining

1 ml of meiotic culture was suspended in Tris 0.25 M, 70% EtOH. 100 μl was spun down and resuspended in 50 μl DDW with 2 μl of DAPI (4',6-diamidino-2-phenylindole, dihydrochloride, InvitroGene). Cells were incubated at 37°C for 10 minutes and visualized under a fluorescence microscope. 200 cells from every time point were characterized and the different cell species (mononucleates, binucleates, tetranucleates) were scored.

### ChIP-on-chip and data analysis

We followed the ChIP-on-chip protocol described by Ren *et al *[45]. Briefly, 50 ml samples were taken at different time points from the meiotic culture. For Sum1-myc meiosis- we sampled from the 15 hours time point, representing mostly late phase binding. Sampling also included a minority of cells from earlier time points due to asynchrony of the culture (see DAPI staining results, Additional File 10). Nevertheless, ChIP-on-chip is sensitive enough to capture targets which are bound only in a subpopulation of the culture, as has been done previously with unsynchronized yeast vegetative cultures [46]. Chromatin was cross-linked by formaldehyde (1%) and ChIP was performed using 10 micrograms of mouse anti myc 9E11 monoclonal antibody (Santa-Cruz). Immunoprecipitated DNA was cleaned by the PCR cleanup system (Promega) and then amplified by the LM-PCR method. During the amplification step Cy5 and Cy3 labeled dUTP were incorporated into the IP enriched and whole cell extract samples, respectively. Labeled samples were purified on Qiaquick PCR purification kit (Qiagen) and hybridized to a spotted glass microarray containing PCR products of all *Saccharomyces cerevisiae *intergenic regions. The arrays were scanned by an Axon-4000B scanner and analyzed using the Axon-pro software. All experiments were performed in duplicate. After Lowess normalization the binding ratio of the duplicates were averaged and a Z score value was calculated for each spot. We chose a threshold of Z > 0.77 and Z > 1.1 for Ndt80 and Sum1, respectively. Both these thresholds correspond to a FDR (false discovery rate) = 0.05 assuming the null Z score distribution to be symmetric around zero [47]. Raw data were deposited in ArrayExpress, accession numbers E-MEXP-1780 and E-MEXP-1779. See Additional File 1 for processed data.

### RNA extraction and labeling for expression profiling

Samples were collected at times 2.5, 5, 7.5, 10, 11, 12, 13, 14, 15, 16, 17, and 18 hours in sporulation medium. For the *sum1Δ *strain samples were collected at 10, 17 and 21 hours and for the MK-ER-Ndt80 strain samples were collected at 0, 8 and 14 hours after the addition of estradiol. Samples were spun at 2,000 rpm for 7 minutes at room temperature, flash frozen in liquid nitrogen and kept at -80°C until RNA extraction. Total RNA was extracted using the RNeasy Midi Kit (Qiagen, Valencia, CA, USA) and 20 μg of RNA were reverse transcribed using superscript II reverse transcriptase (Invitrogen). cDNA products were labeled with Cy3 and Cy5 by the indirect amino-allyl method [48], with minor modifications. Dye incorporation was measured using a spectrophotometer. Reference samples were made from vegetative W303 diploids in YPD.

### Microarray hybridization, scanning and quantification

Double spotted microarrays containing 6240 Yeast ORFs printed as cDNA and more than one hundred control regions (total 6.4 K spots), manufactured by the Genomics Center, University of Toronto, were hybridized according to the TIGR protocol http://pfgrc.tigr.org/protocols/M008.pdf. Equal amounts of both samples (the time course experiments were done in duplicates, using dye swapping), were resuspended in hybridization buffer (5× SSC, 25% formamide, 0.1% SDS, 20 mg yeast tRNA). Samples were incubated for 5 min. at 95º, spun, and put on the slide. The slide was incubated in a 42° water bath overnight, in a hybridization chamber (Corning). The slides were then washed and scanned using an Axon GenePix 4000B scanner (Axon Instruments).

### Data Analysis

Images of scanned arrays were quantified and analyzed using the GenePix Pro 4.1 software (Axon). Data was normalized by Lowess normalization [49]. Poor quality spots were omitted. The final value of every spot is the average of the two available spots for every ORF, if both have passed quality check. All raw data was deposited in ArrayExpress data bank under accession number E-MEXP-1781 for wild-type expression data, E-MEXP-2154 for *sum1Δ *data and E-MEXP-2155 for MK-ER-Ndt80 data.

### De-convolution of wild-type expression data

Since each cell in the culture proceeds into meiosis at a different pace the data in each time point consists of a mixture of cells, each at a slightly different meiotic stage. We collected information about culture synchrony using DAPI staining (Additional File 10) and used it to deconvolve the expression data as described [27], [26]. See Additional File 1 for the deconvulated expression data (log(2) ratios) of all the genes.

### Clustering of expression data

For clustering and comparison with previous work we selected 462 genes whose log fold change between maximum and minimum expression values was higher than 6. Significant overlap (115 genes, p~ 0 using hypergeometric distribution) was found with genes selected by Primig *et al *[13]. K-means clustering with 10 cluster centers was applied to these genes using correlation as the distance metric. The clusters were analyzed for enriched GO annotations using GOLEM [50].

### Analysis of MK-ER-Ndt80 data

The ratio between the relative expression values at late and middle meiosis were calculated for the 140 Ndt80 target genes that show late meiosis repression pattern. In order to quantify the relative contribution of the down regulation of Ndt80 on late meiosis repression, we calculated the following:

In which the weight (W) of the regulation through Ndt80 is a function of the repression seen in the mutant (R_mutant_) and those seen in the wt (R_wt_). The list of the weights for the Ndt80 middle meiosis targets is provided in Additional File 7.

### Binding site scanning

PSSMs were constructed by using the frequency matrices of Ndt80 and Sum1 in the output of WebMotifs. The frequency matrix is converted into weights *W_nuc, j_*:

where *f_nuc,j _*= is the relative frequency of nucleotide *nuc *in position *j*, and *p_nuc _*is the background frequency of the nucleotide *nuc *(in yeast intergenic regions, *p_G _= p_C _= 0.34)*. The PSSM was then used to assign a score *X_i _*to each segment *S*_*i,i*+*len*-1 _of the sequence *S*:

where *nuc*_*i+j-1 *_ε {*A*,*C*,*G*,*T*} is the nucleotide found at position *i+j-1 *of the sequence *S*.

High-scoring segments (large *X_i_*) correspond to putative binding sites for the transcription factors, and the highest score per promoter score is detailed in the results table (Additional File 1). For site determination, we used a threshold of more than 10 for the Ndt80 binding site and more than 12 for the Sum1 binding site. The number of genes passing both thresholds constitutes less than 5% of the genome.

### ChIP-PCR

ChIP was performed in a similar manner to Ren *et al*[45]. Products were cleaned by the PCR cleanup system (Promega). Primers for specific regions (sequence available on request) were designed for radioactive PCR analysis of ChIP products. Input was amplified in a gradient of dilutions in the presence of ^32^P-αdCTP (Amersham), and the IP PCR product was compared to this gradient. PCR fragments were separated on 6% polyacrylamide gels and exposed for autoradiography. Results were quantified using the ImageGauge program (version 3.46, FujiFilm). As a control we used the promoter of *SEC62 *(*YPL093w*).

### Germination and Zymolase assays

Germination assay: Spores of wild-type and *sum1Δ *strains were obtained by incubating diploid cells in SPII (sporulation medium) for 48 hours (according to the protocol of Primig et al [13]). Spores were resuspended at concentration of 10^6 ^cells/ml and 10^5 ^cells/ml and 10 μl drops of this cell suspension were plated on YPD plates. Plates were incubated either at 30ºC or at 37ºC and monitored every 12 hours up to 4 days.

Zymolyase assay: Spores (obtained as above) at a concentration of 10^6 ^cells/ml were incubated at 37° with Zymolase (ICN Biomedicals, USA, 20T, 625 microgram per sample), and 10 μl drops were plated on YEPD plates at indicated times. Plates were incubated at 30°C and monitored every 12 hours up to 4 days.

## Authors' contributions

MK carried out most of the Genomic, molecular and genetic experiments, analyzed the data and drafted the manuscript. ZS carried out the RT-PCR experiments. AG carried out the de-convolution of the genomic expression data. SFA participated in the ChIP-chip data analysis. GZ participated in the expression data analysis. ZBJ participated in the data analysis and in drafting the manuscript. GS supplied materials and facilities, participated in planning the experiments and drafted the manuscript. IS supplied materials and facilities, participated in planning the experiments and drafted the manuscript. All authors have read and approved the final manuscript.

## Supplementary Material

Additional file 1 **Genomic processed data**. The file contains all the genomic (ChIP-chip and expression) data in the different genetic backgrounds, as well as some filters applied to the data, and some validation data from other sources (indicated). Click here for file

Additional file 2 **Validation of microarray data by RT-PCR**. The file contains RT-PCR data on several transcripts in several genetic backgrounds and time points (indicated). Click here for file

Additional file 3 **Genes in 3 binding groups**. The file contains the Y names of the genes in the Ndt80-only, Sum1-only and Common binding groups. Click here for file

Additional file 4 **Validation by ChIP-PCR of selected targets**. The file contains ChIP-PCR results on several promoter regions (indicated). Click here for file

Additional file 5 ***NDT80 *accumulates in ER strain**. The file contains RT-PCR data on the *NDT80 *transcript in WT and ER strains. Shows constitutive expression of *NDT80 *in the presence of Estradiol. Click here for file

Additional file 6 **Kinetics of meiosis in the MK-ER-Ndt80 strain**. The file contains nuclei counting results after DAPI staining in different time points after Estradiol addition to the medium. Click here for file

Additional file 7 **Ndt80 repression metric calculated for every common target gene**. The file contains the calculated metric for the contribution of the indirect regulation of Ndt80 to the repression of every common target gene of Ndt80 and Sum1. Click here for file

Additional file 8 **Sum1 activity during meiosis**. The file contains Western Blot of Sum1-myc during W303 meiosis. Click here for file

Additional file 9 **Strains used in this study**. The file contains the genotypes of all strains used in this study. Click here for file

Additional file 10 **Kinetics of meiosis in WT and sum1-deletion strains**. The file contains nuclei counting results after DAPI staining of WT and sum1-deletion strains during meiosis. Click here for file
