# Supplementary material for: Combination of genomic approaches with functional genetic experiments reveals two modes of repression of yeast middle-phase meiosis genes
Source: BMC Genomics. 2010 Aug 17;11:478. doi: 10.1186/1471-2164-11-478 (PMC3091674; doi:10.1186/1471-2164-11-478)
Supplement: Additional file 2 — Validation of microarray data by RT-PCR. The file contains RT-PCR data on several transcripts in several genetic backgrounds and time points (indicated). [file 1471-2164-11-478-S2.PDF]

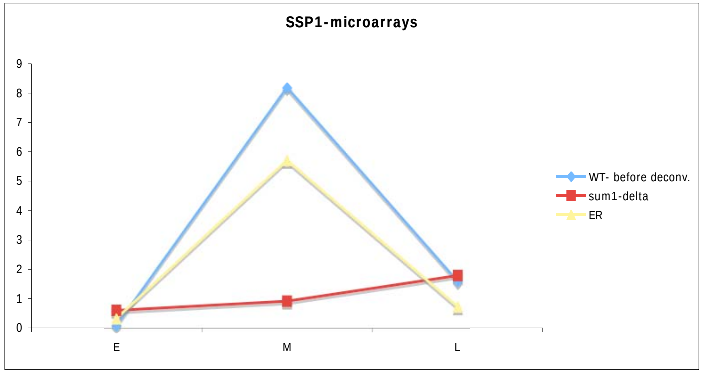

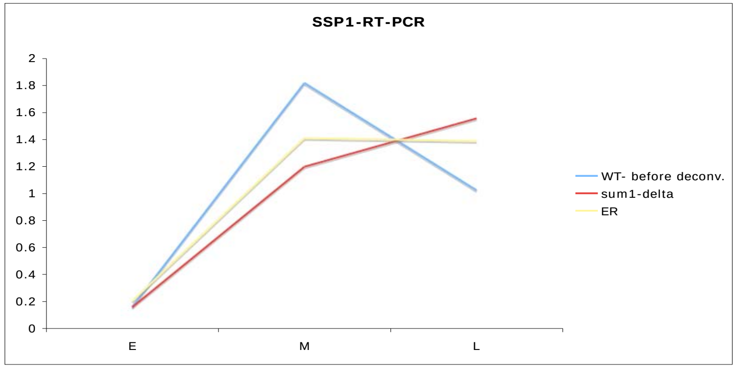

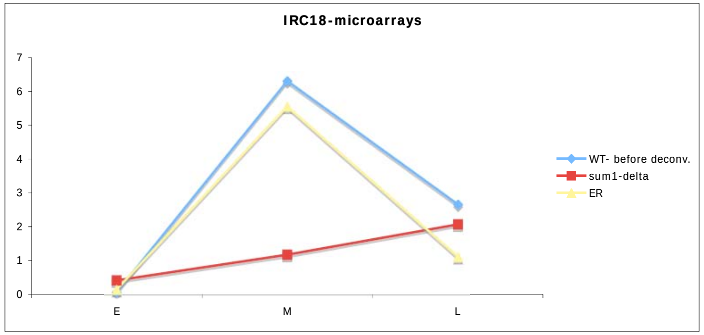

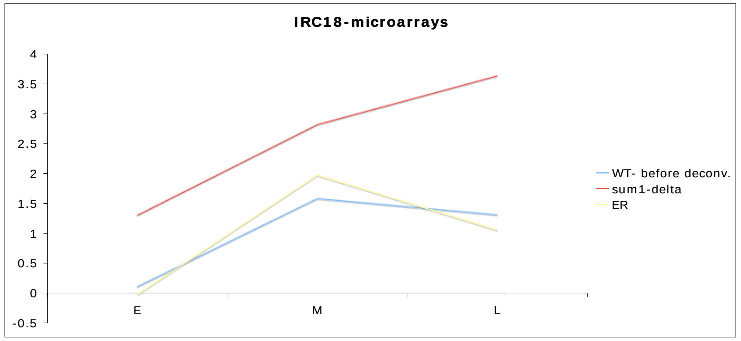

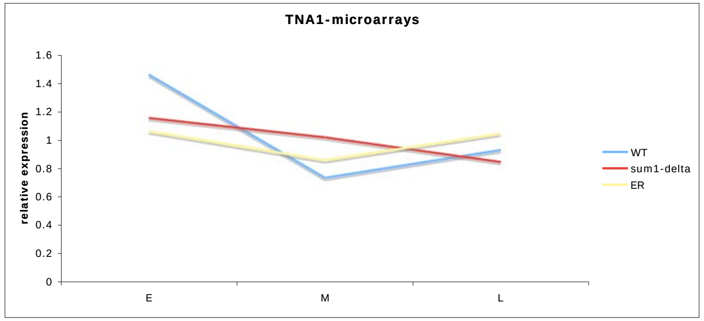

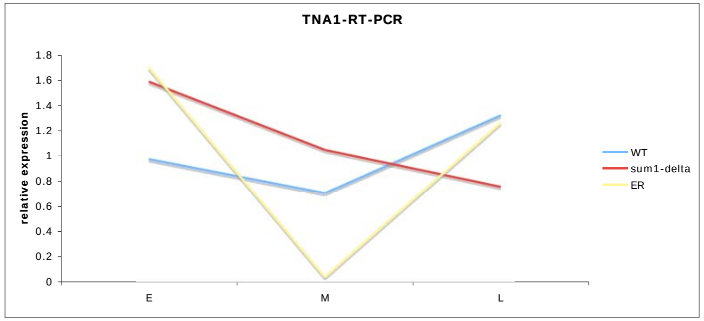

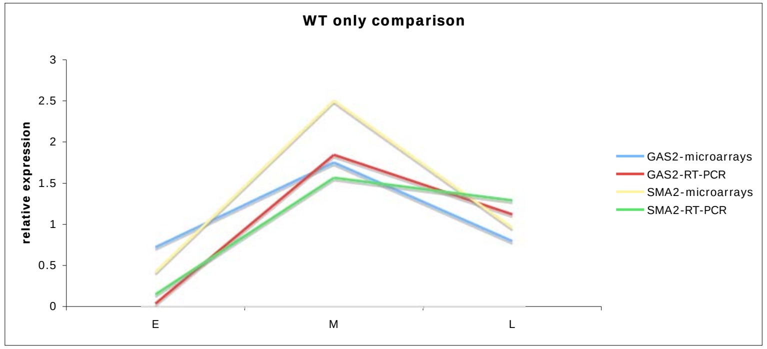

Additional File 2: Validation of microarray data by RT-PCR:

Several transcripts were amplified by RT-PCR (see methods) from the same RNA samples the microarrays were analyzed from. The pattern of expression is the same using RT-PCR and microarrays although actual measured magnitude may differ between the methods.
