# Supplementary material for: Combination of genomic approaches with functional genetic experiments reveals two modes of repression of yeast middle-phase meiosis genes
Source: BMC Genomics. 2010 Aug 17;11:478. doi: 10.1186/1471-2164-11-478 (PMC3091674; doi:10.1186/1471-2164-11-478)
Supplement: Additional file 4 — Validation by ChIP-PCR of selected targets. The file contains ChIP-PCR results on several promoter regions (indicated). [file 1471-2164-11-478-S4.PDF]

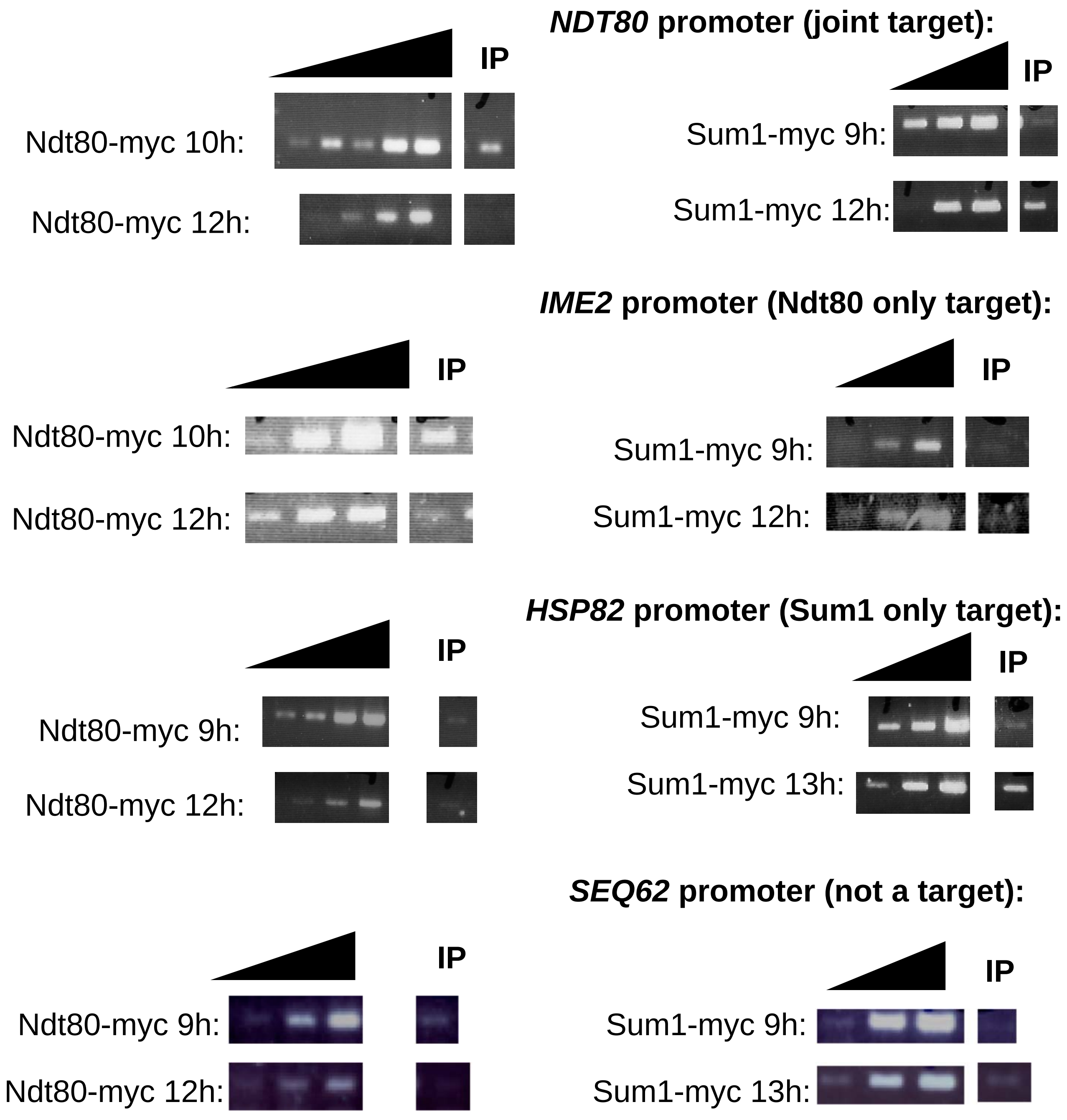

Additional File 4: Validation by ChIP-PCR of selected targets: Samples of ChIP from 10,12 and 13 hours in sporulation medium were analyzed by PCR for the different genes (see Methods). This analysis supports the findings in the ChIP-chip experiment regarding the different groups assignments.
