# Supplementary material for: Combination of genomic approaches with functional genetic experiments reveals two modes of repression of yeast middle-phase meiosis genes
Source: BMC Genomics. 2010 Aug 17;11:478. doi: 10.1186/1471-2164-11-478 (PMC3091674; doi:10.1186/1471-2164-11-478)
Supplement: Additional file 5 — NDT80 accumulates in ER strain. The file contains RT-PCR data on the NDT80 transcript in WT and ER strains. Shows constitutive expression of NDT80 in the presence of Estradiol. [file 1471-2164-11-478-S5.PDF]

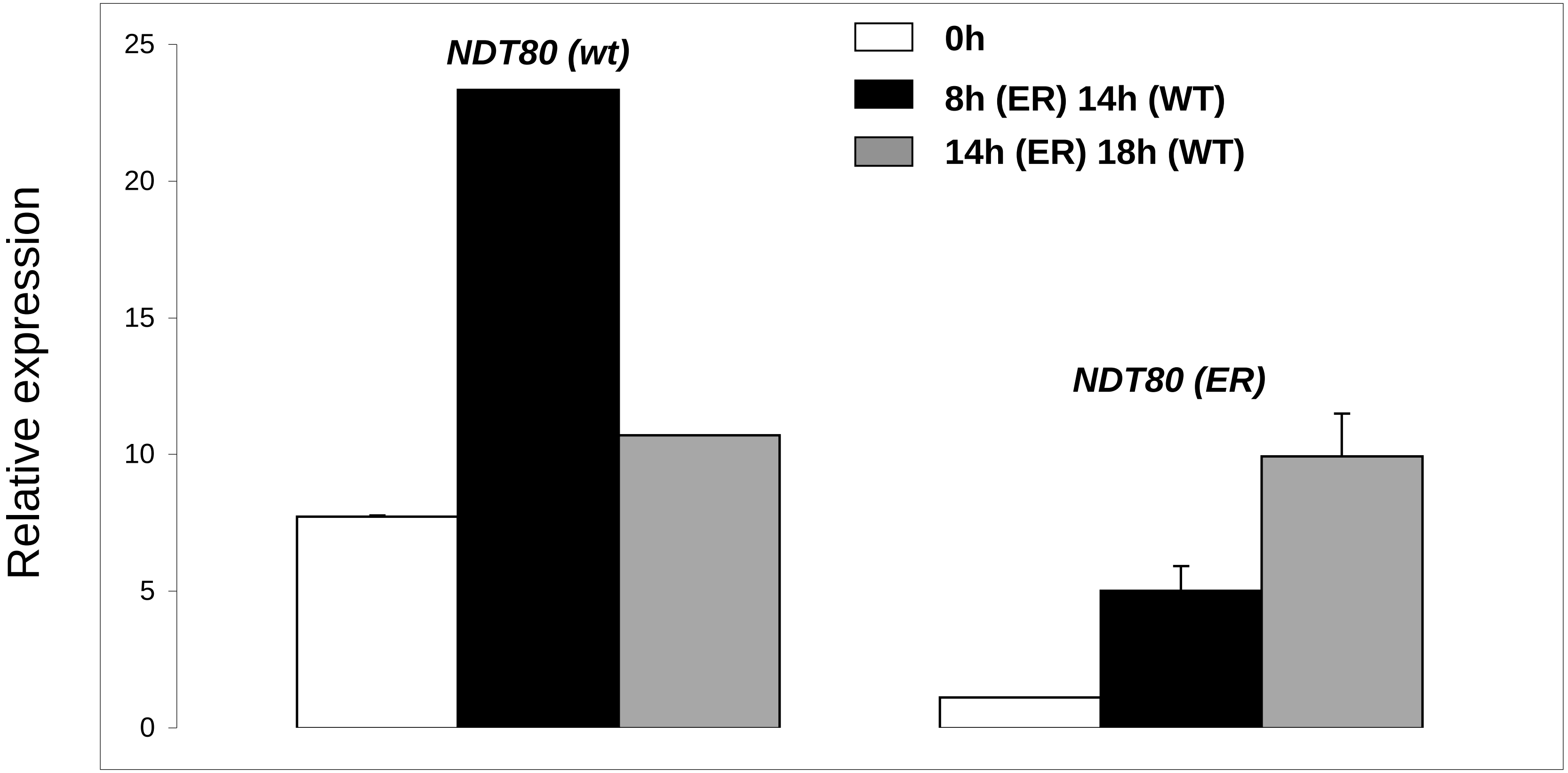

Additional File 5: *NDT80* accumulates in ER strain: RT-PCR of *NDT80* (relative to a control gene, *SEC62*) in WT and the MK-ER-Ndt80 strain (see Methods) at three time points in meiosis. Time points indicated are after the addition of estradiol (ER) or after transfer to sporulation medium (WT). The levels of *NDT80* RNA accumulate in MK-ER-Ndt80.
