# Supplementary material for: Combination of genomic approaches with functional genetic experiments reveals two modes of repression of yeast middle-phase meiosis genes
Source: BMC Genomics. 2010 Aug 17;11:478. doi: 10.1186/1471-2164-11-478 (PMC3091674; doi:10.1186/1471-2164-11-478)
Supplement: Additional file 6 — Kinetics of meiosis in the MK-ER-Ndt80 strain. The file contains nuclei counting results after DAPI staining in different time points after Estradiol addition to the medium. [file 1471-2164-11-478-S6.PDF]

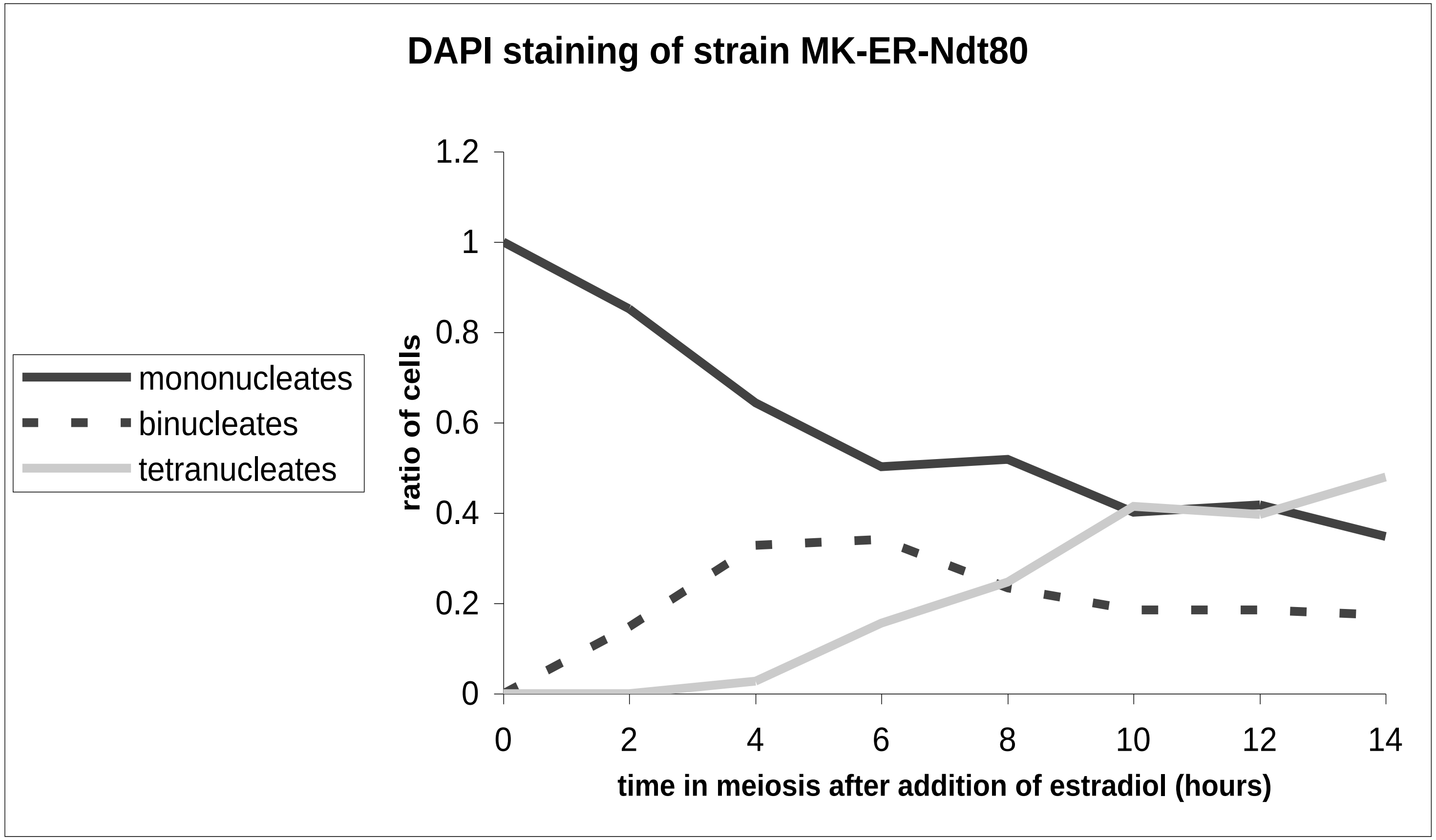

Additional File 6: Kinetics of meiosis in the MK-ER-Ndt80 strain: Cells were taken in every time point and DAPI stained (see Methods). Kinetics show a near wild-type kinetics for meiosis in this strain.
