# Supplementary material for: Combination of genomic approaches with functional genetic experiments reveals two modes of repression of yeast middle-phase meiosis genes
Source: BMC Genomics. 2010 Aug 17;11:478. doi: 10.1186/1471-2164-11-478 (PMC3091674; doi:10.1186/1471-2164-11-478)
Supplement: Additional file 8 — Sum1 activity during meiosis. The file contains Western Blot of Sum1-myc during W303 meiosis. [file 1471-2164-11-478-S8.PDF]

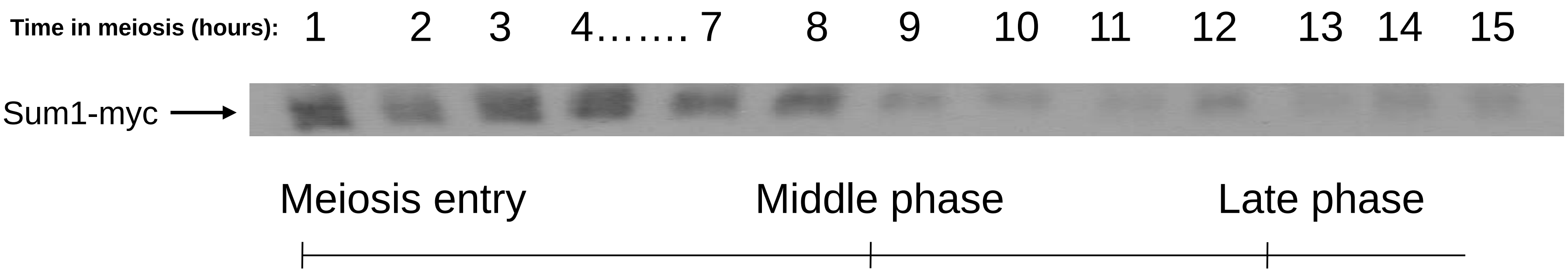

Additional File 8: Sum1 activity during meiosis: Western Blot of Sum1-myc during meiosis. Stages in meiosis according to DAPI staining are indicated. This expression pattern is similar to what was shown for Sum1 protein expression in SK1 cells .
