# Supplementary material for: Combination of genomic approaches with functional genetic experiments reveals two modes of repression of yeast middle-phase meiosis genes
Source: BMC Genomics. 2010 Aug 17;11:478. doi: 10.1186/1471-2164-11-478 (PMC3091674; doi:10.1186/1471-2164-11-478)
Supplement: Additional file 9 — Strains used in this study. The file contains the genotypes of all strains used in this study. [file 1471-2164-11-478-S9.DOC]

Strains used in this study:

| Strain | Genotype |
| --- | --- |
| MKWT | *MATa/MATα*, *ura3/ura3, leu2/leu2, ade2/ade2, his3/his3, trp1/trp1, can1^r^/ can1^r^.* |
| MK1615 | *MATa/MATα*, *ura3/ura3, leu2/leu2, ade2/ade2, his3/his3, trp1/trp1, can1^r^/ can1^r^,*  *NDT80-myc9:TRP1/ NDT80-myc9:TRP1.* |
| MK1613 | *MATa/MATα*, *ura3/ura3, leu2/leu2, ade2/ade2, his3/his3, trp1/trp1, can1^r^/ can1^r^,*  *SUM1-myc9:TRP1/ SUM1-myc9:TRP1*. |
| MKsumdel | *MATa/MATα*, *ura3/ura3, leu2/leu2, ade2/ade2, his3/his3, trp1/trp1, can1^r^/ can1^r^*  *NDT80-myc9:TRP1/ NDT80-myc9:TRP1*, *sum1Δ::URA3*/ *sum1Δ::URA3* |
| MK-ER-Ndt80 | *MATa/MATα*, *ura3/ura3, leu2/leu2, ade2/ade2, his3/his3, trp1/trp1, can1^r^/ can1^r^, ura3::* P_GPD1_-GAL4(848).ER::URA3*/ ura3::* P_GPD1_-GAL4(848).ER::URA3, *ndt80:: TRP1*::pGAL-*NDT80/ ndt80:: TRP1*::pGAL-*NDT80* |
