# Supplementary material for: Combination of genomic approaches with functional genetic experiments reveals two modes of repression of yeast middle-phase meiosis genes
Source: BMC Genomics. 2010 Aug 17;11:478. doi: 10.1186/1471-2164-11-478 (PMC3091674; doi:10.1186/1471-2164-11-478)
Supplement: Additional file 10 — Kinetics of meiosis in WT and sum1-deletion strains. The file contains nuclei counting results after DAPI staining of WT and sum1-deletion strains during meiosis. [file 1471-2164-11-478-S10.PDF]

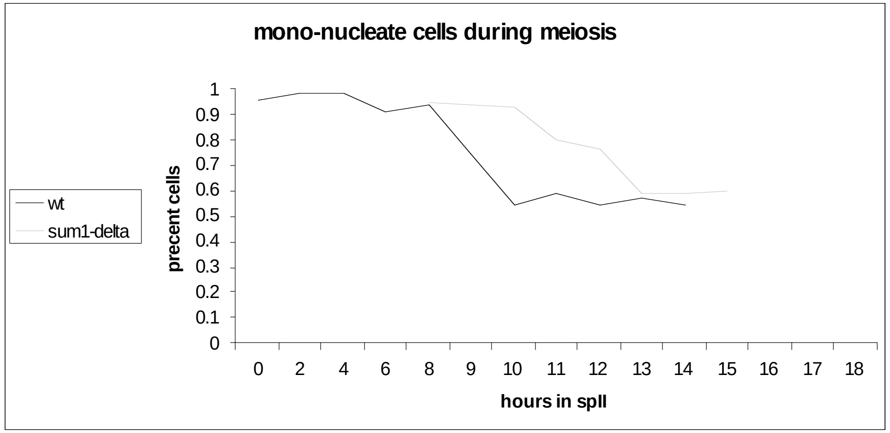

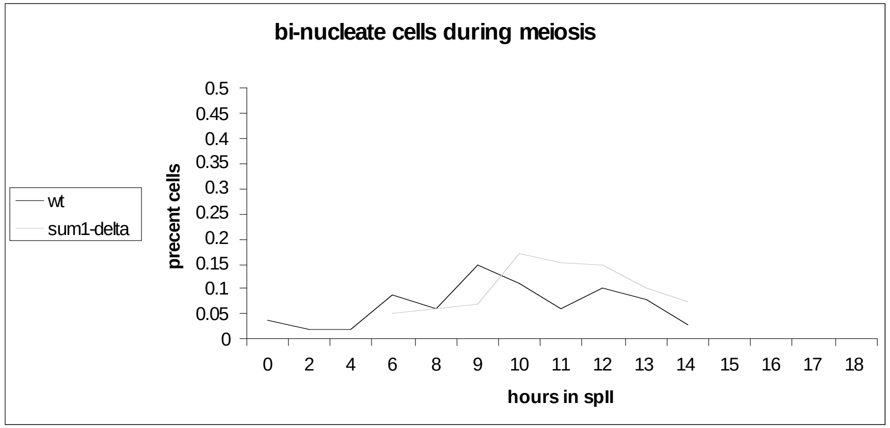

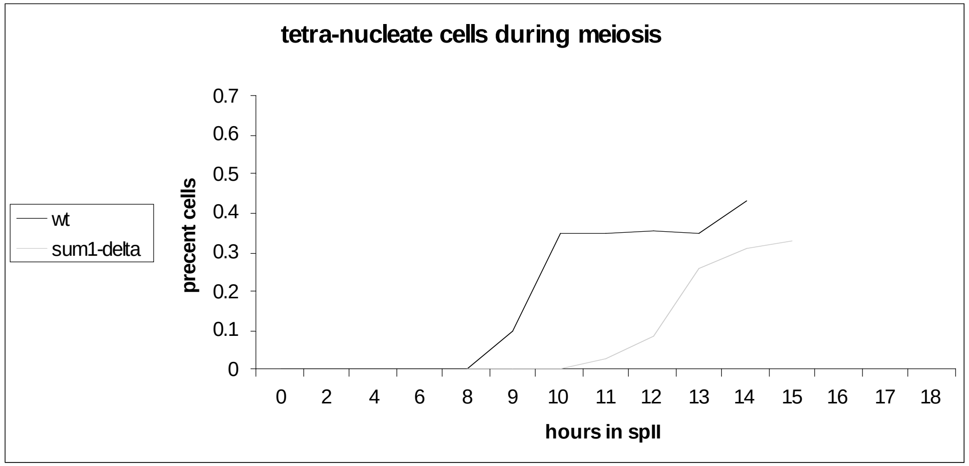

Additional File 10:Kinetics of meiosis in wild-type and sum1-deletion strains: Cells from the expression profile experiment were taken in every time point and DAPI stained (see Methods). Kinetics show that the meiosis in the sum1-deletion strain is slower than that in the WT strain. This data was used for the de-convolution (see Methods). The same analysis was performed on the Ndt80-myc and Sum1-myc strains used for the ChIP on chip experiments and the results were almost identical to those of the WT strain.
